# A Curious Bit of Folklore

**Published:** 1907-09-14

**Authors:** 


### The Practitioner's Relaxations and Hobbies.

### A CURIOUS BIT OF FOLKLORE.

Many medical men may find it a pleasant relaxation from their work to collect scraps of folklore. Those practising in remote country districts have many opportunities of indulging in this pastime; mine have been limited, for the most part, to a town near a large river, yet even here I occasionally come across some curious relics of old beliefs.

### Folklore of the Ebb Tide.

It was asserted by Aristotle that no animal dies near the sea except at the ebbing of the tide. This idea in some form or another has been popular for centuries. Shakespeare makes the Hostess say in describing Falstaff's death, "a' parted even just between twelve and one, even at the turning o' the tide." A commentator, Dr. W. Aldis Wright, observes regarding this passage: "It was and probably still is a popular belief that a person at point of death will live till the tide turns." Such a notion, I find, still exists at Gravesend. An old salt, who used to sit by a dying man in an ale-house on the shore of the Thames, told me he noticed my patient was always worse at the turn of the tide, and then got better after the tide had turned.

Readers of Dickens cannot forget the account in "David Copperfield" of Mr. Barkis "going out with the tide."

"'He's going out with the tide,' said Mr. Peggotty to me, behind his hand.

"My eyes were dim, and so were Mr. Peggotty's; but I repeated in a whisper, 'With the tide?'

"'People can't die along the coast,' said Mr. Peggotty, 'except when the tide's pretty nigh out. They can't be born, unless it's pretty nigh in—not properly born till flood. He's a going out with the tide. It's ebb at half-after three, slack water half-an-hour. If he lives 'till it turns, he'll hold his own 'till past the flood, and go out with the next tide.'

. . . . .

"I was on the point of asking him if he knew me, when he tried to stretch out his arm, and said to me, distinctly, with a pleasant smile:

"'Barkis is willin'!'

"And it being low water, he went out with the tide."

According to Aristotle and Mr. Peggotty, it is at the ebbing of the tide that death always occurs. Dickens probably found his idea to be prevalent among the fishermen when he was at Yarmouth. But at Gravesend, I am told, it does not matter whether the tide is at the ebb or flow; it is just at the turn of the tide that death occurs. "I have often seen it happen, sir," an old shrimper said to me quite recently.

### Time and Tide.

I once heard a lecturer on Shakespeare's works who came to Gravesend make some interesting remarks on the meaning of the word "tide" in the passage already quoted, "a' parted even just between twelve and one, even at the turning o' the tide." He said it would be natural for his hearers, living as they did close to a great river and observing the tides daily, to suppose the word "tide" referred to the ebb and flow of the sea, but this was not so. The real meaning of the passage, he maintained, was at the turning of the *time*, or about midnight, the word "tide" often signifying time formerly, as in the affixes of the old words "shrovetide" and "eventide." Just after midnight, he added, or between twelve and one, it is sometimes supposed the vital powers are depressed.

### Shakespeare's Use of the Word.

But if we examine about forty other passages in which Shakespeare uses the word "tide," we cannot help noting that it is nearly always in the sense of the flux and reflux of the sea, or else of a stream or current. The following passage is a good example: "The tide of blood in me hath proudly flowed in vanity till now: now doth it turn and ebb back to the sea" (2 Hen. IV. v. 2). Also "And think how such an apprehension may turn the tide of fearful faction" (1 Hen. IV., iv. 1).

In some plays we meet with "tide" as "the approaching tide" (Tempest, v. 1), or "the varying tide" (Antony and Cleopatra, i. 4), or "the waxing tide" (Titus Andronicus, iii. 1), or "the swelling tide" (King John, ii. 1). The fact that there is still a belief among dwellers by the Thames that a person dying there will live till the tide turns is in favour, I think, of the view that the Hostess, in her description of Falstaff's death, alluded to the turning of the tide in the river.

The tavern in which Falstaff dies is stated in the play of Henry V. to be in London, and it is thought most likely that it was the Boar's Head in Eastcheap which Shakespeare had in his mind. This old tavern was situated at only a little distance from the side of the Thames.

### "Born at the Tide."

As regards the tides having any influence on the time of our birth, I have never in the whole course of my experience as a medical practitioner heard a hint or suggestion made to that effect. But it has been said that monthly nurses on the Welsh coast sometimes inquire, "How goes the tide?"

In ancient times there was a deep-rooted belief in a mysterious correspondence and sympathy between our bodies and the outer world. The moon, "the governess of floods," as Shakespeare calls her, and "the moist star upon whose influence Neptune's empire stands," was supposed to regulate by her "age" or changes the period of births and deaths, and now and then we have evidence of this kind of moon-lore lingering among us still.

